# Vitamin B_12_-Containing Plant Food Sources for Vegetarians

**DOI:** 10.3390/nu6051861

**Published:** 2014-05-05

**Authors:** Fumio Watanabe, Yukinori Yabuta, Tomohiro Bito, Fei Teng

**Affiliations:** Division of Applied Bioresources Chemistry, The United Graduate School of Agricultural Sciences, Tottori University, Tottori 680-8553, Japan; E-Mails: yabuta@muses.tottori-u.ac.jp (Y.Y.); D12A3003M@edu.tottori-u.ac.jp (T.B.); D13A3003Z@edu.tottori-u.ac.jp (F.T.)

**Keywords:** cobalamin, dried purple laver, nori, vitamin B_12_ deficiency

## Abstract

The usual dietary sources of Vitamin B_12_ are animal-derived foods, although a few plant-based foods contain substantial amounts of Vitamin B_12_. To prevent Vitamin B_12_ deficiency in high-risk populations such as vegetarians, it is necessary to identify plant-derived foods that contain high levels of Vitamin B_12_. A survey of naturally occurring plant-derived food sources with high Vitamin B_12_ contents suggested that dried purple laver (nori) is the most suitable Vitamin B_12_ source presently available for vegetarians. Furthermore, dried purple laver also contains high levels of other nutrients that are lacking in vegetarian diets, such as iron and *n*-3 polyunsaturated fatty acids. Dried purple laver is a natural plant product and it is suitable for most people in various vegetarian groups.

## 1. Introduction

**Figure 1:**
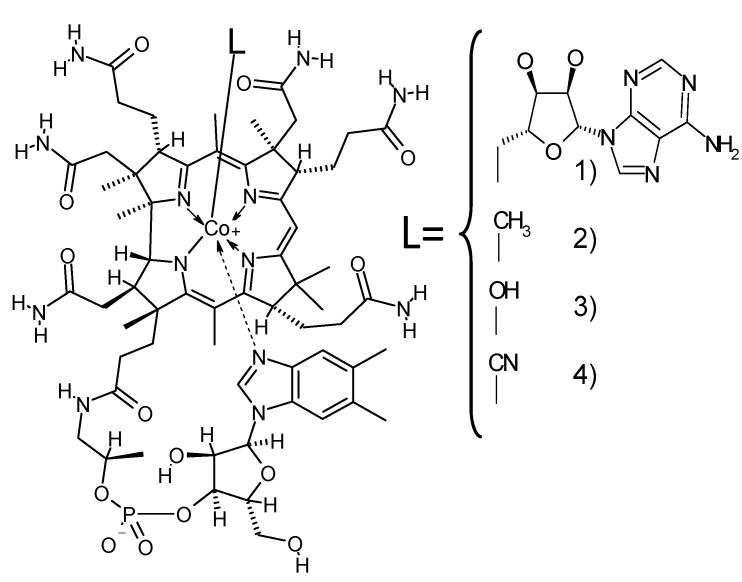
Structural formula of Vitamin B_12_ and partial structures of Vitamin B_12_ compounds. The partial structures of the Vitamin B_12_ compounds only show the regions of the molecule that differ from Vitamin B_12_. (**1**) 5′-Deoxyadenosylcobalamin; (**2**) methylcobalamin; (**3**) hydroxocobalamin; and (**4**) cyanocobalamin or Vitamin B_12_.

Vitamin B_12_ (molecular weight = 1355.4) belongs to the “corrinoids” group, which comprises compounds that contain a corrin macrocycle. The term “Vitamin B_12_” is usually restricted to cyanocobalamin, which is the most chemically stable and unnatural form of cobalamin [1], but Vitamin B_12_ refers to all potentially biologically active cobalamins in the present review. Cyanocobalamin is included in most human dietary supplements, and it is readily converted into the coenzyme forms of cobalamin, *i.e.*, methylcobalamin functions as a coenzyme for methionine synthase (EC 2.1.1.13; involved in methionine biosynthesis), and 5′-deoxyadenosylcobalamin functions as a coenzyme for methylmalonyl-CoA mutase (EC 5.4.99.2; involved in amino acid and odd-chain fatty acid metabolism in mammalian cells) [2,3] (Figure 1). Corrinoids with a base other than 5,6-dimethylbenzimidazole as the lower ligand (cobalt-coordinated nucleotide) were recently found in certain foods and they are inactive in humans [4]. 

Vitamin B_12_ is synthesized only by certain bacteria, and it is primarily concentrated in the bodies of predators located higher in the food chain [5]. Vitamin B_12_ is well-known to be the sole vitamin that is absent from plant-derived food sources. Foods (meat, milk, eggs, fish, and shellfish) derived from animals are the major dietary sources of Vitamin B_12_ [4]. The recommended dietary allowance (RDA) of Vitamin B_12_ for adults is set at 2.4 μg/day in the United States (and Japan) [6,7]. The major signs of Vitamin B_12_ deficiency are megaloblastic anemia and neuropathy [6]. Vegetarians are at a higher risk of Vitamin B_12_ deficiency than non-vegetarians [8]. The frequencies of the deficiency among vegetarians were estimated as 62%, 25%–86%, 21%–41%, and 11%–90% in pregnant women, children, adolescents, and elderly subjects, respectively, by review of the 18 reports evaluating Vitamin B_12_ status of vegetarians [9]. The objective of this review is to present up-to-date information on Vitamin B_12_-containing plant-derived food sources to prevent vegetarians from developing Vitamin B_12_ deficiency.

## 2. Main Types of Vegetarian Diets

There are several main types of vegetarian groups: (1) Lacto-ovo vegetarianism [10]: many people are familiar with this type of vegetarianism, which comprises most vegetarians. “Lacto” indicates that a person consumes milk and milk products (butter, yogurt, cheese, *etc.*), and “ovo” means that a person consumes eggs. In general, lacto-ovo vegetarians do not consume animal meats (including fish and shellfish). Some vegetarian groups are ovo only or lacto only, *i.e.*, they consume only eggs or only milk and its products, respectively, as animal products; (2) Raw veganism [11]: this diet is mostly or entirely based on fresh fruits, vegetables, nuts, and seeds; (3) Fruitarianism [12]: this is generally a raw style of eating that primarily depends on fruits, nuts, and seeds; (4) Buddhist vegetarianism [13]: this is a vegan diet that excludes all animal products and Allium family vegetables (onion, garlic, leeks, and shallots) on ethical grounds; (5) Macrobiotic [14]: this diet is primarily focused on grains, beans, and similar staples, including some vegetables and other whole foods. Processed foods and most animal products are strongly avoided; and (6) Jain vegetarianism [15]: another religious dietary practice that includes dairy products, but excludes eggs and honey as well as root vegetables.

## 3. Nutritional Characterization of Vegetarian Diets

From a nutrient intake perspectives, vegetarian diets are usually rich in carbohydrates, *n*-6 polyunsaturated fatty acids, dietary fibers, carotenoids, folic acid, Vitamin C, Vitamin E, and magnesium (Mg), but these diets are relatively low in proteins, saturated fatty acids, *n*-3 polyunsaturated fatty acids (particularly eicosapentaenoic and docosahexaenoic acids), Vitamin A (retinol), Vitamin B_12_, Vitamin D_3_ (chlolecalciferol), zinc, iron, and calcium [16,17,18] (Table 1). In particular, Vitamins A, B_12_, and D_3_ are found only in animal-derived foods, whereas Vitamin D_2_ (ergocalciferol) and provitamin A (β-carotene) are found in mushrooms and vegetables, respectively [19,20]. Furthermore, Vitamin D_3_ can be synthesized in the human skin under sunlight [21]. A vegetarian diet usually provides a low intake of saturated fatty acids and cholesterol but a high intake of dietary fibers and health-promoting phytochemicals (e.g., various polyphenol compounds) due to an increased consumption of fruits, vegetables, whole-grains, legumes, nuts, and various soy products. As a result, vegetarians typically have lower body mass index, serum cholesterol levels, and blood pressure [18]. Compared with non-vegetarians, vegetarians also have reduced rates of mortality due to ischemic heart disease, probably because of lower blood cholesterol. However, there are no clear differences with respect to other major causes of death such as stroke and cancers [17]. Craig [17] reported that, compared with non-vegetarians, vegetarians have lower incidences of hypertension, stroke, type 2 diabetes, and certain cancers. Pawlak *et al.* [9] showed that vegetarians can develop Vitamin B_12_ depletion or deficiency regardless of their demographic characteristics, place of residency, age, or type of vegetarian diets. The Vitamin B_12_ content is not high in whole eggs (approximately 0.9–1.4 μg/100 g), most of which is located in the egg yolk [22]. The average bioavailability of Vitamin B_12_ from cooked eggs is 3.7%–9.2% [23]. Thus, the Vitamin B_12_ in eggs is generally poorly absorbed compared with that in other animal-derived products [24]. The Vitamin B_12_ content of various types of milk is very low (approximately 0.3–0.4 μg/100 g) [4], and appreciable losses of Vitamin B_12_ occur during the processing of milk [25,26]. Approximately 20%–60% of the Vitamin B_12_ that is initially present in milk is recovered in cottage cheese, hard cheese, and blue cheese [27]. The Vitamin B_12_ content in the whey is considerably reduced during lactic acid fermentation [28]. These observations explain why Vitamin B_12_ deficiency is relatively common in lacto-ovo-vegetarians. Furthermore, food-bound Vitamin B_12_ malabsorption occurs with certain gastric dysfunctions, particularly atrophic gastritis with low stomach acid secretion [29]. The body storage level of Vitamin B_12_ is significantly depleted by a persistent vegetarian diet; thus Vitamin B_12_ deficiency may readily develop in elderly vegetarians. However, Vitamin B_12_ deficiency may go undetected in vegetarians because their diets are rich in folic acid, which may mask vitamin B_12_ deficiency until severe health problems occur [30]. Vitamin B_12_ deficiency contributes to the development of hyperhomocysteinemia, which is recognized as a risk factor for atherothrombotic [31] and neuropsychiatric disorders [32], thereby negating the beneficial health effects of a vegetarian lifestyle. Thus, many investigators have suggested that vegetarians should maintain an adequate intake of Vitamin B_12_ by consuming supplements that contain Vitamin B_12_ or Vitamin B_12_-fortified foods [29,33].

**Table 1:** Nutrient imbalance in vegetarian diets.

Rich	Low
Fiber	Vitamin A
Vitamin C	Vitamin D_3_
Vitamin E	Vitamin B_12_
Folate	Iron
Magnesium	Cholesterol
*n*-6 Polyunsaturated fatty acids	*n*-3 Polyunsaturated fatty acids
Carbohydrates	Saturated fatty acids

## 4. Vitamin B_12_-Containing Plant-Derived Food Sources

In the United States, ready-to-eat cereals fortified with Vitamin B_12_ comprise a high proportion of the dietary Vitamin B_12_ intake [6]. Several research groups have suggested that eating a breakfast cereal fortified with folic acid, Vitamins B_12_ and B_6_ increases the blood concentrations of these vitamins and decreases the total homocysteine concentrations in the plasma of elderly subjects [34]. Thus, Vitamin B_12_-fortified breakfast cereals may be a particularly valuable source of Vitamin B_12_ for vegetarians. However, processed foods are strongly avoided by most vegetarians in addition to animal products. Thus, it is necessary to identify plant-derived food sources that naturally contain a large amount of Vitamin B_12_ to prevent Vitamin B_12_ deficiency in vegetarians.

### 4.1. Vitamin B_12_-Enriched Beans and Vegetables Produced Using Organic Fertilizers or Hydroponics

Mozafar [35] demonstrated that adding an organic fertilizer such as cow manure significantly increased the Vitamin B_12_ content of spinach leaves, *i.e.*, approximately 0.14 μg/100 g fresh weight. However, the consumption of several hundred grams of fresh spinach would be insufficient to meet the RDA of 2.4 μg/day for adult humans [6,7]. Furthermore, our recent [36] and unpublished research indicates that most organic fertilizers, particularly those made from animal manures, contain considerable amounts of inactive corrinoid compounds. These compounds are also present in human feces where they account for more than 98% of the total corrinoid content [37].

Some researchers attempted to prepare Vitamin B_12_-enriched vegetables by treating them with a solution that contains high levels of Vitamin B_12_ [38,39]. This resulted in significant increases in the plant Vitamin B_12_ contents, thereby suggesting that Vitamin B_12_-enriched vegetables may be particularly beneficial to vegetarians. However, artificially Vitamin B_12_-enriched vegetables may not fit the philosophy of vegetarians. 

### 4.2. Fermented Beans and Vegetables

The Vitamin B_12_ contents of soybeans are low or undetectable. However, a fermented soybean-based food called tempe contains a considerable amount of Vitamin B_12_ (0.7–8.0 μg/100 g) [40]. Bacterial contamination during tempe production may contribute to the increased Vitamin B_12_ content of tempe [41]. Other fermented soybean products contain minute amounts of Vitamin B_12_ [42,43].

Only trace amounts of Vitamin B_12_ were found in broccoli, asparagus, Japanese butterbur, mung bean sprouts, tassa jute, and water shield [44]. Fermented Korean vegetables (kimuchi) contain traces (<0.1 μg/100 g) of Vitamin B_12_ [43]. High Vitamin B_12_ (approximately 10 μg/100 g)-enriched vegetable products tend to be produced by fermentation with certain lactic acid or propionic bacteria [45,46].

Vitamin B_12_ is found in various types of tea leaves (approximately 0.1–1.2 μg Vitamin B_12_ per 100 g dry weight) [47]. For example, Vitamin B_12_-deficient rats were fed a Japanese fermented black tea (Batabata-cha) drink (50 mL/day, equivalent to a daily dose of 1 ng Vitamin B_12_) for 6 weeks, and the urinary methylmalonic acid excretion (an index of Vitamin B_12_ deficiency) levels in the tea drink-supplemented rats was significantly lower than in those of the deficient rats [48]. These results indicate that Vitamin B_12 _found in fermented black tea is bioavailable in rats. However, the consumption of 1–2 L of the fermented tea drink (typical regular consumption in Japan), which is equivalent to 20–40 ng of Vitamin B_12_, is not sufficient to meet the RDA of 2.4 μg/day for adult humans.

### 4.3. Edible Mushrooms

Several wild edible mushroom species are popular among vegetarians in European countries. Zero or trace levels (approximately 0.09 μg/100 g dry weight) of Vitamin B_12_ were measured in the dried fruiting bodies of porcini mushrooms (*Boletus* sp.), parasol mushrooms (*Macrolepiota procera*), oyster mushrooms (*Pleurotus ostreatus*), and black morels (*Morchella conica*). In contrast, the fruiting bodies of black trumpet (*Craterellus cornucopioides*) and golden chanterelle (*Cantharellus cibarius*) contained higher levels of Vitamin B_12_ (1.09–2.65 μg/100 g dry weight) than the abovementioned mushrooms [49]. To determine whether the fruiting bodies of dried black trumpet and golden chanterelle contain Vitamin B_12_ or other corrinoid compounds that are inactive in humans, we purified the corrinoid compound using an immunoaffinity column and identified it as Vitamin B_12_ by liquid chromatography-electrospray ionization tandem mass spectrometry [49]. In addition, high levels of Vitamin B_12_ were detected in the commercially available dried shiitake mushroom fruiting bodies (*Lentinula edodes*), which are used in various vegetarian dishes. The Vitamin B_12_ contents of dried shiitake mushroom fruiting bodies (100 g dry weight) significantly varied and the average Vitamin B_12_ value was approximately 5.61 μg [50]. Dried shiitake mushroom fruiting bodies rarely contained the inactive corrinoid, Vitamin B_12_[*c*-lactone] as well as Vitamin B_12 _[50]. Lion’s mane mushroom (*Hericium erinaceus*) fruiting bodies also contain considerable amounts of Vitamin B_12_[*c*-lactone] [51]. Stabler *et al.* [52] demonstrated that Vitamin B_12_[*c*-lactone] binds very weakly to the most specific Vitamin B_12_-binding protein, *i.e.*, the intrinsic factor involved in the gastrointestinal absorption of Vitamin B_12_, and it strongly inhibits Vitamin B_12_-dependent enzymes, methylmalonyl-CoA mutase and methionine synthase.

The consumption of approximately 50 g of dried shiitake mushroom fruiting bodies could meet the RDA for adults (2.4 μg/day), although the ingestion of such large amounts of these mushroom fruiting bodies would not be possible on a daily basis.

### 4.4. Edible Algae

**Figure 2:**
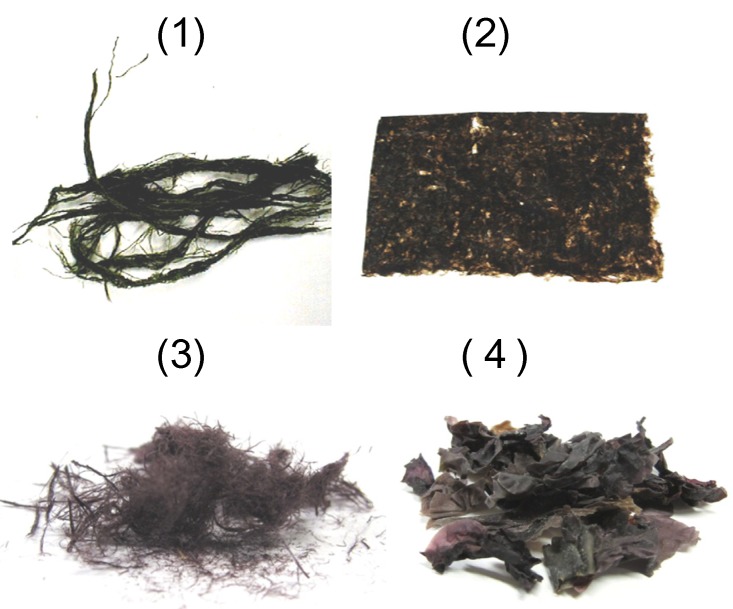
Various types of dried green and purple lavers are Vitamin B_12_ sources: (**1**) a Japanese green laver, (Suji-aonori, *Entromopha prolifera*); (**2**) ordinary purple lavers (*Porphyra* sp.; nori, which has been formed into a sheet and dried); (**3**) Taiwan purple laver (Hong-mao-tai, *Bangia atropurpurea*); and (**4**) New Zealand purple laver (Karengo, a mixture of *Porphyra cinnamomea* and *Porphyra virididentata*).

Various types of edible algae are consumed worldwide as food sources. Dried green laver (*Enteromorpha* sp.) and purple laver (*Porphyra* sp.) are the most widely consumed edible algae, and they contain substantial amounts of Vitamin B_12_ (approximately 63.6 μg/100 g dry weight and 32.3 μg/100 g dry weight, respectively) [53] (Figure 2). However, excluding these two genera, other edible algae contain zero or only traces of Vitamin B_12_ [54]. To determine whether dried purple and green lavers contain Vitamin B_12_ or inactive corrinoids, the algal corrinoid compounds were purified and confirmed as Vitamin B_12_ [55,56]. A substantial amount (133.8 μg/100 g dry weight) of Vitamin B_12_ was found in dried Korean purple laver (*Porphyra* sp.), but seasoned and toasted laver products contain lower amounts of Vitamin B_12_ (approximately 51.7 μg/100 g dry weight) [57]. In particular, when the dried purple laver was treated by toasting until the laver’s color changed from purple to green, the decreases in the Vitamin B_12_ contents of the seasoned and toasted laver products were not due to the loss or destruction of Vitamin B_12_ during the toasting process [57]. *In vitro* gastrointestinal digestion experiments indicated that the estimated digestion rate of Vitamin B_12_ from dried purple laver was approximately 50% at pH 2.0 (as a model of normal gastric function). The release of free Vitamin B_12_ from the purple laver significantly decreased to approximately 2.5% at pH 7.0 (as a model of severe atrophic gastritis) [57]. Edible purple laver predominantly contains coenzyme forms (5′-deoxyadenosylcoblamin and methylcobalamin) of Vitamin B_12_ or hydroxocobalamin (or both) [57,58,59].

To measure the biological activity of Vitamin B_12_ in lyophilized purple laver (*Porphyra yezoensis*), the effects of laver feeding were investigated in Vitamin B_12_-deficient rats [58]. Urinary methylmalonic acid excretion was undetectable within 20 days of initiating a diet supplemented with dried purple laver (10 μg of Vitamin B_12_/kg diet), and the hepatic Vitamin B_12_ (especially coenzyme Vitamin B_12_) levels significantly increased. These results indicate that Vitamin B_12_ obtained from purple laver is bioavailable in rats. A nutritional analysis of six vegan children who had consumed vegan diets including brown rice and dried purple laver (nori) for 4–10 years suggested that the consumption of nori may prevent Vitamin B_12_ deficiency in vegans [60]. Our preliminary study indicated that similar dried purple laver products that are available in local markets in Taiwan (Hong-mao-tai, *Bangia atropurpurea*) and New Zealand (Karengo, a mixture of *P. cinnamonea* and *P. virididentata*) contained 28.5 ± 3.9 and 12.3 ± 1.9 μg of Vitamin B_12_ per 100 g weight, respectively (Figure 2).

For a long time, it was unclear whether algae have an absolute requirement for Vitamin B_12_ for growth, and why algae that lack a requirement of Vitamin B_12_ for growth contain substantial amounts of Vitamin B_12_. However, recent biochemical and bioinformatics studies have accurately defined the Vitamin B_12_ requirements of various algae (half of all algal species absolutely require Vitamin B_12_ for their growth), and they have suggested possible physiological functions for Vitamin B_12_ in algae [61,62].

Furthermore, the standard tables of food composition in Japan [63] indicate that dried purple laver (per 100 g) contains various other nutrients that are lacking in vegetarian diets, such as Vitamin A (3600 μg of Vitamin A equivalent as provitamin A), iron (10.7 mg), and *n*-3 polyunsaturated fatty acids (1.19 g), as well as Vitamin B_12_ (77.6 μg). Purple laver also contains a large amount of a pigment protein, phycoerythrin, which is digested in the intestine to release the covalently linked chromophore moiety, a phycoerthrobilin compound (a potent antioxidant) [64].

**Figure 3:**
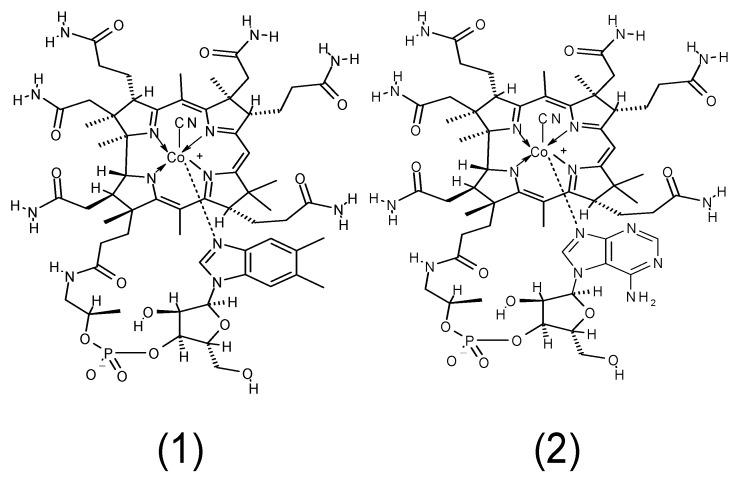
Structural formulae of Vitamin B_12_ and pseudovitamin B_12_. (**1**) Vitamin B_12_ and (**2**) pseudovitamin B_12_ (7-adeninyl cyanocobamide).

*Chlorella* tablets (eukaryotic microalgae *Chlorella* sp.) used in human food supplements contain biologically active Vitamin B_12_ [65]. However, our unpublished study indicates that the Vitamin B_12_ contents significantly differ among various commercially available *Chlorella* tablets (from zero to several hundred μg of Vitamin B_12_ per 100 g dry weight); we do not have any information on why such a huge variation occurs. Thus, vegetarians who consume *Chlorella* tablets as a source of Vitamin B_12_ should check the nutrition labeling of *Chlorella* products to confirm their Vitamin B_12_ contents. High levels of Vitamin B_12_ are described in the nutritional labels of dietary supplements that contain edible cyanobacteria such as *Spirulina*, *Aphanizomenon*, and *Nostoc*. However, although substantial amounts of Vitamin B_12_ were detected in these commercially available supplements using a microbiological Vitamin B_12_ assay method, these supplements often contained large amounts of pseudovitamin B_12_ [66,67,68,69,70,71] (Figure 3), which is biologically inactive in humans. Therefore, edible cyanobacteria and their products are not suitable for use as sources of Vitamin B_12_ for vegetarians.

## 5. Conclusions

**Figure 4:**
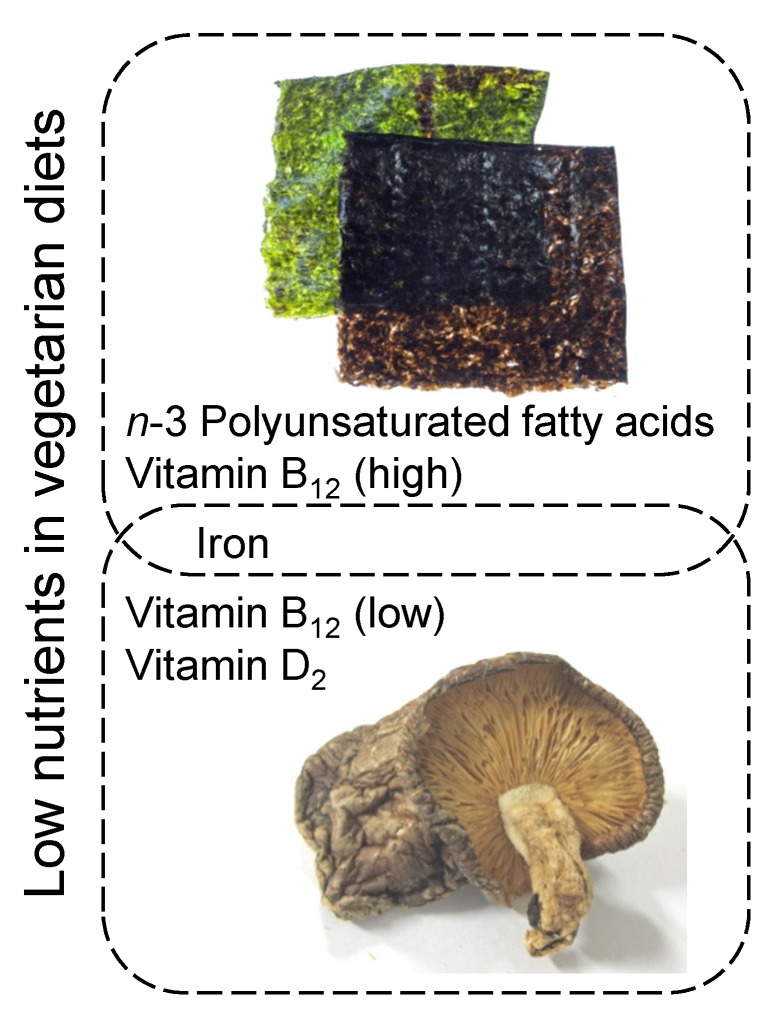
Proposed method for improving nutrient imbalance in vegetarian diets using dried purple laver as a Vitamin B_12_ source in addition to other plant-based food sources.

A survey of naturally occurring and high Vitamin B_12_-containing plant-derived food sources showed that nori, which is formed into a sheet and dried, is the most suitable Vitamin B_12_ source for vegetarians presently available. Consumption of approximately 4 g of dried purple laver (Vitamin B_12_ content: 77.6 μg /100 g dry weight) supplies the RDA of 2.4 μg/day. In Japan, several sheets of nori (9 × 3 cm^2^; approximately 0.3 g each) are often served for breakfast. A large amount of nori can be consumed as certain forms of sushi (vinegared rice rolled in nori). In particular, hand-rolled sushi made by wrapping rice and fillings with nori is easy to prepare and facilitates the consumption of a large amount of nori. When dried purple laver was treated by toasting until the laver’s color changed from purple to green, the toasting treatment did not affect the Vitamin B_12_ contents [57]. Dried purple lavers could also be a suitable food item for integration in Italian, French, and other forms of western cuisine. Dried purple laver is also a rich source of iron and *n*-3 polyunsaturated fatty acids (Figure 4). Dried purple laver is a natural plant product; therefore, it is suitable for most vegetarian groups. Among edible mushrooms, relatively high levels of Vitamin B_12_ were detected in the commercially available shiitake mushroom fruiting bodies, but the Vitamin B_12_ content significantly varies (1.3–12.7 μg/100 g dry weight), which is significantly lower than that found in dried purple laver. However, the dried shiitake mushroom fruiting bodies (per 100 g) contain 18.9 mg of Vitamin D_2_ (ergocalciferol) and 2.0 mg of iron [63], which are also nutrients that vegetarian diets tend to lack. Thus, the use of these plant-based food sources can significantly improve the nutrient imbalance in vegetarian diets to reduce the incidence of Vitamin B_12_ deficiency in vegetarians.
